# A simple sequence repeats marker of disease resistance in shrimp *Litopenaeus vannamei* and its application in selective breeding

**DOI:** 10.3389/fgene.2023.1144361

**Published:** 2023-07-27

**Authors:** Bin Yin, Haiyang Wang, Shaoping Weng, Sedong Li, Jianguo He, Chaozheng Li

**Affiliations:** ^1^ State Key Laboratory of Biocontrol, Southern Marine Science and Engineering Guangdong Laboratory (Zhuhai), School of Marine Sciences, School of Life Sciences, Sun Yat-sen University, Guangzhou, China; ^2^ Guangdong Provincial Key Laboratory of Marine Resources and Coastal Engineering, Guangdong Provincial Key Laboratory for Aquatic Economic Animals, Guangzhou, China; ^3^ China-ASEAN Belt and Road Joint Laboratory on Marine Aquaculture Technology, Guangzhou, China; ^4^ Guangdong Evergreen Feed Industry Co., Ltd., Zhanjiang, China; ^5^ Maoming Branch, Guangdong Laboratory for Lingnan Modern Agriculture, Maoming, China

**Keywords:** shrimp, simple sequence repeat (SSR), selective breeding, disease resistance, IRF

## Abstract

The polymorphism of the simple sequence repeat (SSR) in the 5′ untranslated coding region (5′-UTR) of the antiviral gene *IRF* (*LvIRF*) has been shown to be implicated in the resistance to viral pathogens in shrimp *Litopenaeus vannamei* (*L. vannamei*). In this study, we explored the potential of this (CT)*n*-SSR marker in disease resistance breeding and the hereditary property of disease resistance traits in offspring. From 2018 to 2021, eight populations were generated through crossbreeding by selecting individuals according to microsatellite genotyping. Our results demonstrated that shrimp with the shorter (CT)*n* repeat exhibited higher resistance to white spot syndrome virus (WSSV) or Decapod iridescent virus 1 (DIV1); meanwhile, these resistance traits could be inherited in offspring. Interestingly, we observed that the longer (CT)*n* repeats were associated with bacterial resistance traits. Accordingly, shrimp with longer (CT)*n* repeats exhibited higher tolerance to *Vibrio parahaemolyticus* infection. Taken together, these results indicate that the single (CT)*n*-SSR marker could be used to selective breeding for both resistance to virus and bacteria in shrimps.

## 1 Introduction

Microsatellites, also known as simple sequence repeats (SSRs) or short tandem repeats (STRs), were first coined by Litt and Luty in 1989 (Litt and Luty, 1989), which are simple repeated motifs consisting of 1–6 base pairs found in both coding and non-coding regions. The advantage of SSRs as genetic markers is that they are inherited as co-dominant markers in a Mendelian fashion. Furthermore, high polymorphism rates and a broad distribution throughout the genome have made SSRs one of the popular genetic markers for use in breeding programs (Wright and Bentzen, 1994; Morgante et al., 2002; Gous et al., 2013). In fact, genetic markers have been widely applied in the breeding of economically important crops such as rice (Xue et al., 2018). By analysing the agronomic traits of rice varieties throughout the world, a number of SSRs have been obtained for marketable traits, such as grain size (GLW7/OsSPL13) (Si et al., 2016), chilling tolerance (COLD1) (Ma et al., 2015), disease resistance (Pigm) (Deng et al., 2017) and nitrate-use efficiency (NITR1.1) (Hu et al., 2015). SSR-assisted breeding has also been reported in aquatic animals. In shrimp *Penaeus monodon*, two specific SSR DNA fingerprints were present in considerably higher frequencies in the white spot syndrome virus (WSSV) susceptible shrimp population (Chakrabarty et al., 2015). In addition, eight IFN system genes were identified as anti-disease molecular markers for resistance breeding of gibel carp by transcriptome sequencing analysis (Mou et al., 2018).

Shrimp *Litopenaeus vannamei* is a worldwide aquaculture species, and the total of yield exceeds 4 million tons per year. However, various shrimp diseases are responsible for huge losses, especially WSSV-caused white spot syndrome (WSS) (Flegel, 2012). To date, there is no effective method for preventing or controlling WSSV infection in shrimp, but selective breeding of the WSSV-resistant population could be effective. In shrimp, innate immunity plays a key role in the defence against a wide variety of pathogenic invasions, such as bacteria, fungi and viruses. Notably, the IRF-Vago-JAK/STAT pathway, which is similar to the IRF-IFN-JAK/STAT pathway in vertebrates, has been functionally identified as playing a significant role in defence against multiple viruses, including WSSV (Li et al., 2015a). In this cascade, the *L. vannamei* interferon regulatory factor (*LvIRF*) can be activated during viral infection and then translocates to the nucleus to initiate the expression of the *L. vannamei Vago4* (*LvVago4*) gene (Li et al., 2015a). *LvVago4* is an arthropod cytokine encoding a viral-activated secreted peptide that inhibits viral infection by activating the JAK-STAT axis (Chen et al., 2011; Wang and He, 2019). Previously, we have shown that a microsatellite with a variable number of CT repeats presents in the 5′ untranslated coding region (5′-UTR) of *LvIRF*, and the length of the (CT)*n* can influence the expressional of *LvIRF* (Yin et al., 2019). As a result, the SSR in LvIRF (LvIRF-SSR) can be used as a molecular marker to selectively breed a new generation of WSSV-resistant shrimp.

In this study, we uncovered that shrimp with shorter (CT)*n* repeats in the 5′-UTR of *LvIRF* exhibited high tolerance to viruses, such as WSSV and decapod iridescent virus 1 (DIV1), while shrimp with longer (CT)*n* repeats in the 5′-UTR of *LvIRF* exhibited high tolerance to *Vibrio parahaemolyticus* (VP). We used LvIRF-SSR as a genetic marker for the breeding programme of shrimp *L. vannamei*. Through selecting individuals for cross-matching through microsatellite genotyping and artificial challenge for comparing resistance to virus and bacteria in offspring, we demonstrated that the LvIRF-SSR could be used in *L. vannamei* breeding to target improvement of antiviral and antibacterial traits.

## 2 Materials and methods

### 2.1 Microsatellite genotyping

As previously described in our study, the CT microsatellite from the *LvIRF* 5′-UTR region was genotyped using FAM fluorogenic probes to discriminate allele size (Yin et al., 2019). Primers (Table 1) were designed to amplify a 202 bp fragment containing 18 CT repeats that were used as a reference, and deviation from this size allowed us to deduce the number of (CT)*n* repeats for different alleles. Samples were sequenced and analyzed using an ABI Genetic Analyser 3730 XL. The alleles were divided into two groups: short (S) alleles were ≤ 18 repeats, and long (L) alleles were > 18 repeats, based on the median as previously described (Yin et al., 2019).

**TABLE 1:** Primers used in this study.

Names	Sequences (5′–3′)	Size/bp	Tm/°C
Quantitative RT-PCR			
LvIRF-F	ACG​CTG​CCC​TCT​TTC​GCT​AC	162	60
LvIRF-R	ACG​CTG​TGA​ACC​TGA​AGT​ATC​G		
LvEF-1α-F	GTA​TTG​GAA​CAG​TGC​CCG​TG	143	60
LvEF-1α-R	ACC​AGG​GAC​AGC​CTC​AGT​AAG		
PCR for genotyping			
LvIRF-5′UTR-F	FAM-ATCGGGATCCACTCGCAGATAC	202	56
LvIRF-5′UTR-R	GGC​GAC​CTT​AGA​CCG​ACG​AGT​T		

### 2.2 Experimental animals

**FIGURE 1:**
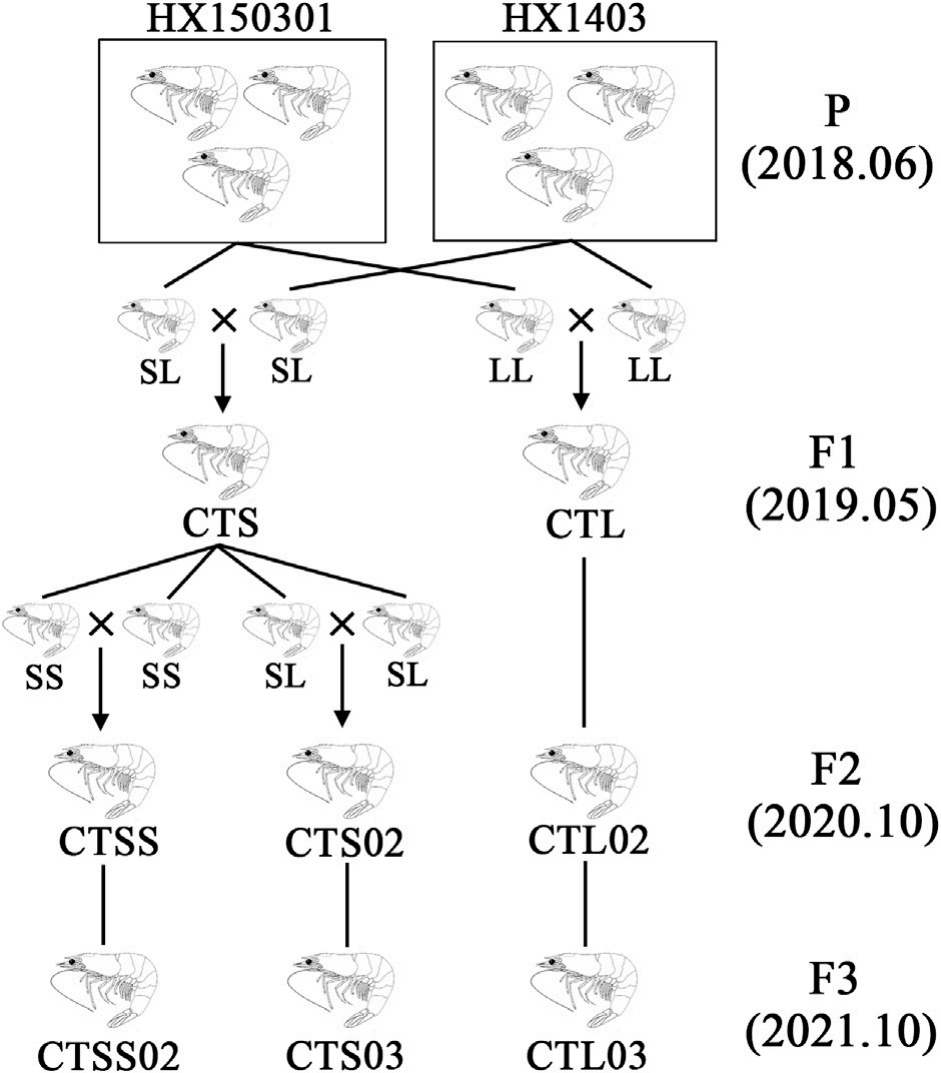
Schematic representation of the selective population. The HX150301 and HX1403 populations as founder stocks, with LvIRF-SSR genotyping used to select individuals as parents for cross-matching to obtain F1 in 2019. Based on F1, the same method was used for breeding F2 and F3 in 2020 and 2021, respectively. The homozygous populations CTSS and CTSS02, as well as the heterozygous populations CTS02 and CTS03, were separated from CTS with LvIRF-SSR genotyping.

The *L. vannamei* breeding programme was carried out at the Hengxing shrimp farm in Zhanjiang City, China. The HX150301 and HX1403 populations served as the founder stocks, and two populations were stocked in F1. One population, known as the CTS, had parents with short CT repeats (S) in the *LvIRF* 5′-UTR, while the other population had parents with only long CT repeats (L), and was known as the CTL population. On the basis of F1, the same method was used for breeding F2 and F3 (Figure 1). To investigate the differences in the disease-resistant traits between F2 and F3 populations, a total of 400 shrimps were collected from each population, and 100 shrimps were received the treatment of WSSV, DIV1 and *V. parahaemolyticus*, as well as PBS as a control. The shrimp’s body weight was 6.0 ± 1.0 g each, and each population of shrimp was cultured in filtered sea water with 2.5% salinity at 26°C in a recirculating water tank and fed with fodder at a rate of 5% of body weight per day.

### 2.3 Pathogens

The WSSV isolate (accession No. AF440570.1) originated from a batch of WSSV-infected *L. vannamei* collected in Zhanjiang in 2021, and the DIV1 isolate (accession No. NC_055165.1) originated from a batch of DIV1-infected *L. vannamei* collected in Maoming in 2021. WSSV and DIV1 were prepared from shrimp muscle tissue previously infected with WSSV or DIV1 and stored at −80°C. The muscle tissue was homogenised to prepare a WSSV inoculum at a final concentration of about 1 × 10^6^ virions/50 μL and a DIV1 inoculum with a final concentration of about 1 × 10^5^ virions/50 μL, which was injected into each shrimp, respectively (Li et al., 2014; Liao et al., 2020). All tanks were checked every 4 h, and dead shrimp were collected and marked. All shrimp were collected and marked on the fifth day (120 h) after infection to calculate cumulative mortality. All experimental materials were stored at −20°C with DNA and RNA holders (TakaRa, Dalian, China) for downstream DNA and RNA extractions.

The bacteria *V. parahaemolyticus* (VP) is sourced from Guangdong Microbiological Collection Center (GDMCC No. 1.306). VP was cultured in Luria broth (LB) medium overnight at 37°C, and the bacteria were harvested by centrifugation (5,000 g, 5 min) and washed twice in phosphate buffer saline (PBS) to remove growth medium. In a previous study, a final injection density of VP was adjusted to yield approximately 1 × 10^5^ CFU/50 μL (Li et al., 2017). On the 5th day (120 h) after infection, all shrimp were collected and marked to estimate cumulative mortality.

### 2.4 Quantitative RT-PCR

Total RNA was extracted using the RNeasy Mini Kit (Qiagen, Hilden, Germany) and reverse transcribed into cDNA using the PrimeScript RT reagent kit (TakaRa, Dalian, China). Quantitative RT-PCR was performed to analyze *LvIRF* gene expression, and the *LvEF-1α* gene was detected as an internal control.

### 2.5 Statistical analysis

All data are presented as means ± SD. Student *t*-test was used to calculate the comparisons between groups of numerical data. Using GraphPad Prism software, cumulative mortality data were subjected to statistical analysis to generate the Kaplan ± Meier plot (log-rank χ^2^ test).

A test for goodness-of-fit analysis was performed to determine whether the alleles were inherited in a Mendelian fashion. The equation used with the respective degrees of freedom (*df*) was $\chi^{2}=\sum_{i=1}^{k}{\left(O-E\right)}^{2}/E$ , *df* = k-1, where O is the observed number of individuals in the genotypic class, E is the expected number of individuals in the same genotypic class and k is the number of genotypic classes (Eguiarte, 1998).

Associated analysis using 2 × 2 contingency tables and Fisher’s exact test. Bonferroni corrections were applied to account for the multiple testing of the LvIRF-SSR genotype groups [*p corr < 0.0167 (0.05/3)], following Fisher exact tests (Bush and Moore, 2012).

## 3 Results

### 3.1 Selective breeding of shrimp with *LvIRF* microsatellite marker

The *L. vannamei* breeding programme at Hengxing shrimp farm in Zhanjiang City, China, began in 2018, with a captive population in Hengxing serving as a founder stock. These populations were imported from abroad or domesticated in China for many years. The breeding programme was based on genotyping by the LvIRF-SSR marker to establish multiple families. As previously described in our study (Yin et al., 2019), the HX150301 and HX1403 populations were used as founder stocks, with LvIRF-SSR genotyping used to select individuals as parents for cross-matching. In the CTS population, where the parents contained allele S ((CT)*n* ≤ 18 repeats), the CTL population only had allele L ((CT)*n* > 18 repeats). Based on F1, the same method was used for breeding F2 and F3, and the homozygous population CTSS was separated from CTS in F2 (Figure 1).

### 3.2 LvIRF-SSR genotyping in offspring

**FIGURE 2:**
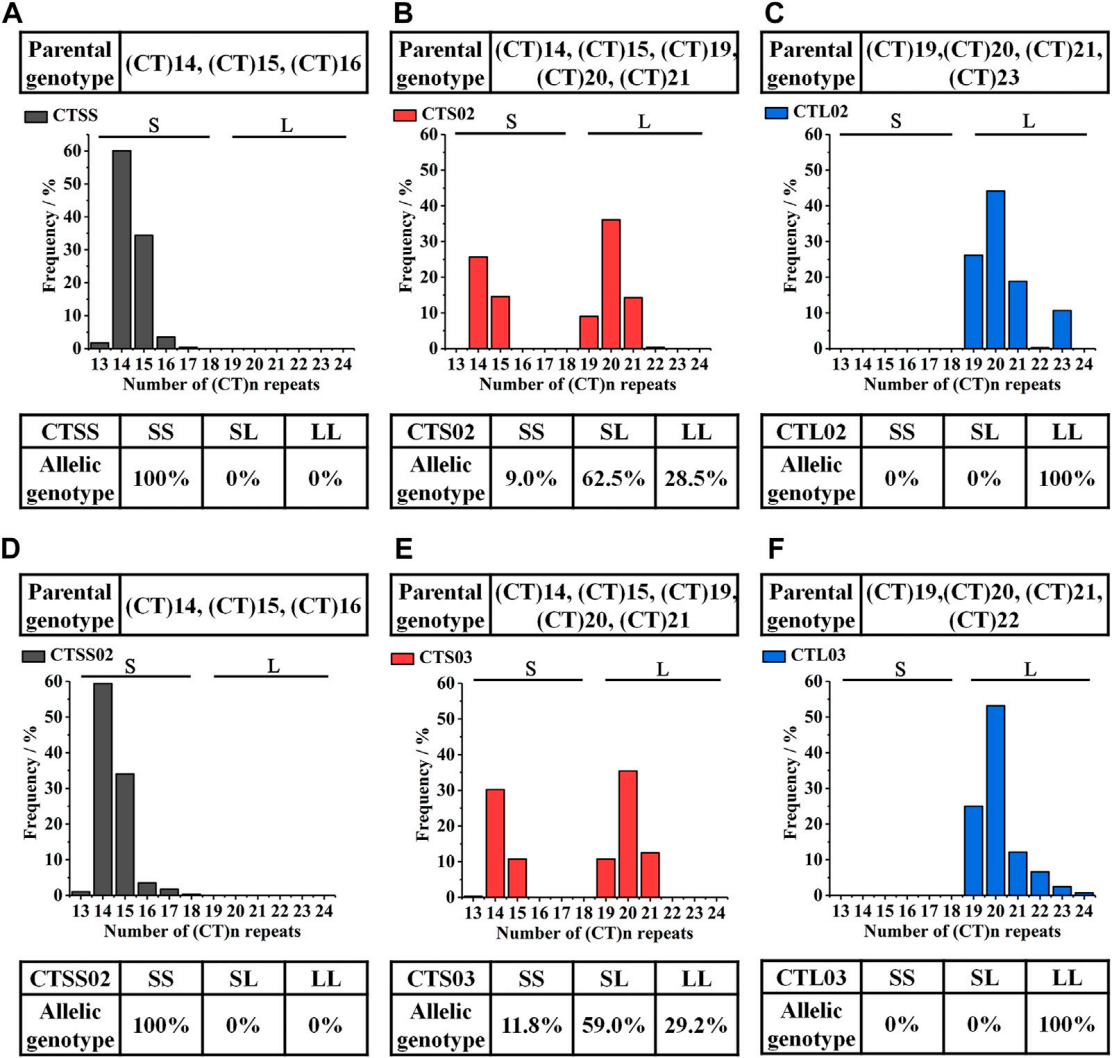
Genotyped of LvIRF-SSR in F2 and F3. **(A–C)** The allelic distribution in F2 three populations: CTSS **(A)**, CTS02 **(B)** and CTL02 **(C)**. **(D–F)** The allelic distribution in F3 three populations: CTSS02 **(D)**, CTS03 **(E)** and CTL03 **(F)**.

Based on the breeding scheme illustrated in Figure 1, we obtained eight new shrimp populations in three generations from 2018 to 2021. LvIRF-SSR was used to genotype each population and count the allele frequencies to validate selection breeding and analysis of Mendelian inheritance. The statistical results of CTS and CTL were shown in previous studies: the CTS population contained the S allele, such as the (CT)14 and (CT)15 genotypes, while the CTL population did not, as expected (Yin et al., 2019). Similar to the genotyping results in F1, in F2 and F3, the homozygous populations CTSS and CTSS02 only contained the S allele of (CT)14 and (CT)15; CTL02 and CTL03 had the L allele of (CT)19, (CT)20, (CT)21, (CT)22 and (CT)23 (Figure 2). As expected, the heterozygous populations CTS02 and CTS03 contained S and L alleles.

The goodness-of-fit test in three heterozygous populations showed that the alleles of LvIRF-SSR did not segregate in a Mendelian ratio of 1:2:1. In the CTS population, the χ^2^ values of 21.90 was higher than 5.99 (*df* = 2), indicating that the heterozygous populations of CTS deviated from the Mendelian ratio of 1:2:1, manifested as homozygous of the LvIRF-SSR genotype SS deletion (Table 2). Homozygous LvIRF-SSR genotype SS deletion in the CTS02 and CTS03 populations also deviated from the Mendelian ratio of 1:2:1 (Table 2).

**TABLE 2:** LvIRF-SSR genotypic frequencies among F1 and F2 populations and analysis of Mendelian inheritance.

Population	Parental genotype		Observed no. of offspring in each genotypic class			Expected ratios	*n*	χ^2^ values^a^
	Male	Female						
CTS	SL	SL	SS	SL	LL	1:2:1	80	21.90
			2	50	28			
CTS02	SL	SL	SS	SL	LL	1:2:1	144	19.89
			13	90	41			
CTS03	SL	SL	SS	SL	LL	1:2:1	144	13.38
			17	85	42			

^a^ χ^2^ values (*p* < 0.05): 5.99 for *df* = 2.

### 3.3 Short CT microsatellite genotypes improve the resistance of shrimps to virus

**FIGURE 3:**
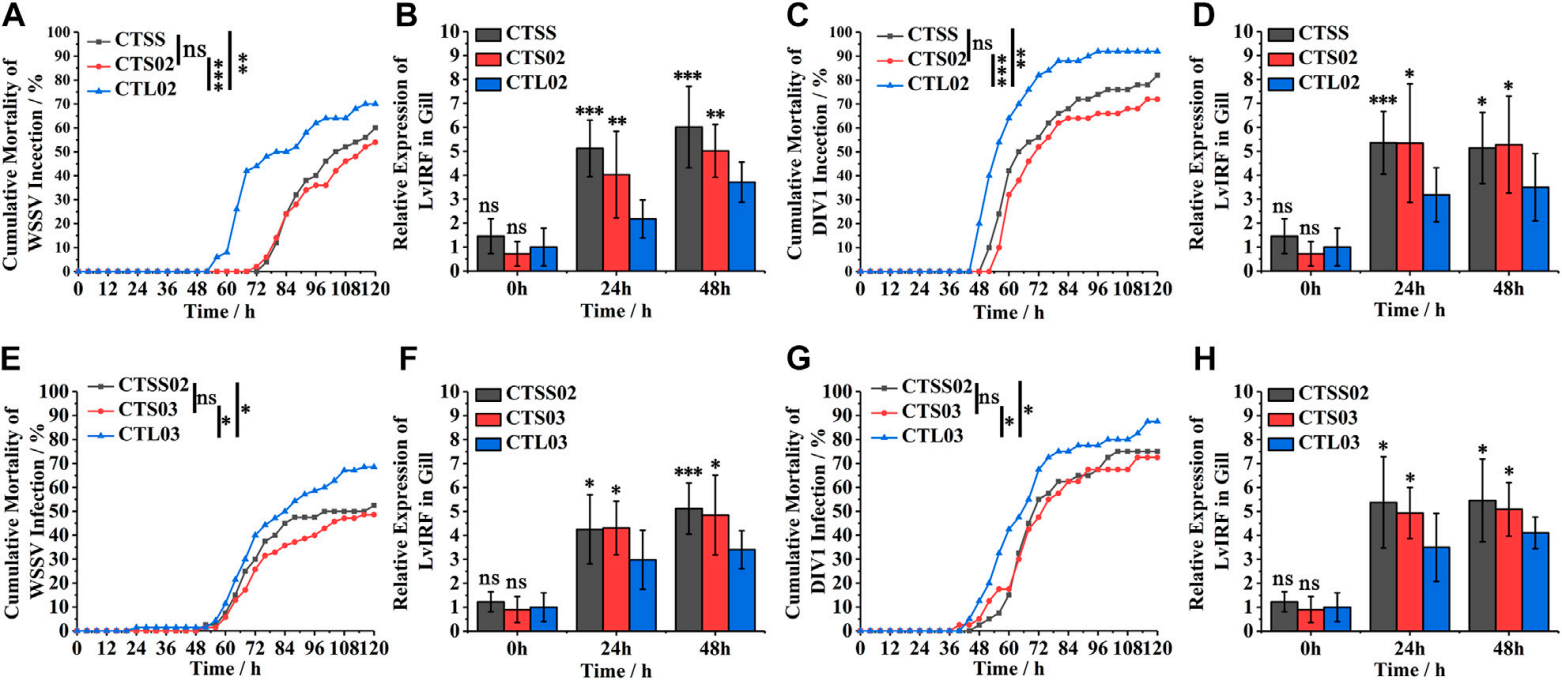
Differences in antiviral traits between shrimp populations in F2 and F3. **(A,C)** Cumulative mortality of F2 populations infected with WSSV and DIV1. **(B,D)** The expression of *LvIRF* in the gill after WSSV and DIV1 infection. **(E,G)** Cumulative mortality of F3 populations infected with WSSV and DIV1. **(F,H)** The expression of *LvIRF* in the gill after WSSV and DIV1 infection. Cumulative mortality data were analyzed statistically by the Kaplan ± Meier plot (log-rank χ^2^ test). *p < 0.05, **p < 0.01, ***p < 0.001, ns, not significant. Quantitative RT-PCR was performed for each sample using the *LvEF-1α* gene as an internal control by the Livak (2^−ΔΔCT^) method. Expression levels in the gill were used to determine the mean fold change (means ± SD, *n* = 9. *p < 0.05, **p < 0.01, ***p < 0.001, ns, not significant.

WSSV and DIV1 were used to compare the resistance to virus in breeding populations. In F2, 120 h after WSSV infection, the cumulative mortality rate of the CTL02 population was 70%, significantly higher than those of CTSS and CTS02 populations (Figure 3A). Furthermore, the cumulative mortality of the CTL02 population was substantially higher than that of the CTS02 and CTL02 populations after 120 h of DIV1 infection (Figure 3C). The *LvIRF* gene expression in gills by quantitative RT-PCR revealed that after 24 h and 48 h of WSSV infection, the expression of *LvIRF* in CTSS and CTS02 populations was significantly higher than that in the CTL02 population, which was 1.85 times and 1.35 times after WSSV infection, respectively (Figure 3B). After DIV1 infection, the expression of *LvIRF* in the CTSS and CTS02 populations was 1.68-fold and 1.51-fold higher than that in the CTL02 population (Figure 3D). The results of the artificial challenge in the F2 generation showed that the resistance to virus of shrimps with the S allele (CTSS and CTS02 populations) were higher than those of the CTL02 population, and the increased expression of LvIRF could significantly improve the antiviral traits of the shrimp population. A similar result was shown in the F3 generation; the CTSS02 and CTS03 populations were resistant to the virus compared to CTL03 (Figures 3E, G), and the expression of *LvIRF* in CTSS02 and CTS03 populations was significantly higher than in CTL02 population after WSSV and DIV1 infection (Figures 3F, H). The above results suggest that the population of shrimp containing short CT genotypes in the 5′-UTR of *LvIRF* were resistant to WSSV and DIV1 infection, thereby improving their survival rate in this assay.

### 3.4 Long CT microsatellite genotypes are associated with VP resistance

**FIGURE 4:**
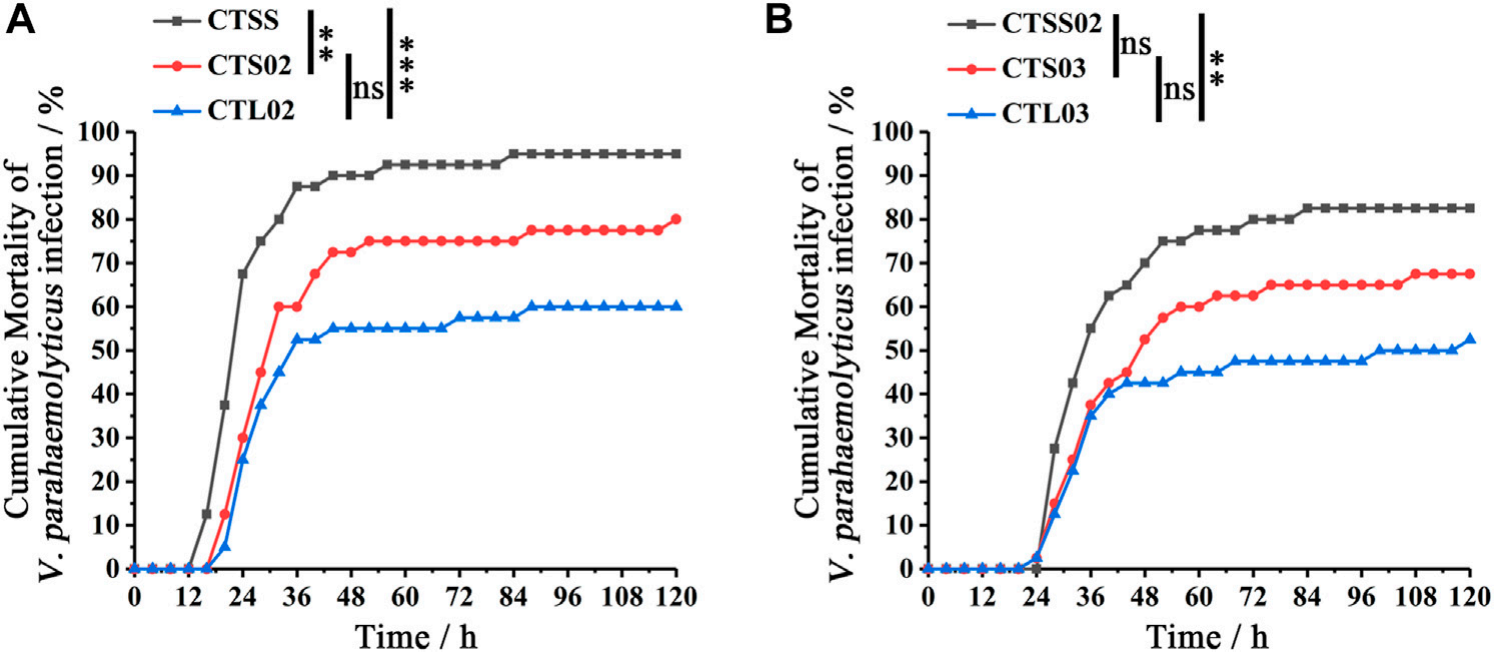
Differences in *Vibrio parahaemolyticus* resistance between shrimp populations in F2 and F3. **(A)** Analysis of *Vibrio parahaemolyticus* resistance differences among the three populations in F2. **(B)** Analysis of VP resistance differences among the three populations in F2. Cumulative mortality data were analyzed statistically by the Kaplan ± Meier plot (log-rank χ^2^ test). **p < 0.01, ***p < 0.001, ns, not significant.

VP was used to compare the resistance to bacteria in breeding populations. In F2, after 120 h of VP infection, the cumulative mortality rate of the CTSS population was close to 100%, significantly higher than the CTS02 and CTL02 populations (Figure 4A), while the CTL02 and CTS02 populations showed no significant difference in the anti-VP trait (*p* = 0.078). A similar result was shown in F3, and the anti-VP trait of the CTL03 population were substantially higher than those of the CTSS02 population (Figure 4A). The CTSS02 and CTS03 populations showed no significant difference in the anti-VP trait (*p* = 0.066), while the CTS03 and CTL03 populations showed no significant difference in the anti-VP trait (*p* = 0.177). After 120 h of VP infection, we divided each shrimp population into two groups based on survival status: dead (D) and surviving (S). Through microsatellite genotyping, we could attribute one of three bi-allelic genotypes to each individual: SS, SL or LL. Fisher’s exact test and Bonferroni corrections showed that SS and LL genotypes were associated with VP resistance. The CTL02 population showed a significant difference in the LL genotype (*p* corr < 0.0003), while the CTSS population showed a significant difference in the SS genotype (*p* corr < 0.0003). Similar to the CTL02 population, the LL genotype associated with VP resistance showed a substantial difference in the CTL03 population (*p* corr < 0.0003), while the SS genotype exhibited a significant difference in the CTSS02 population (*p* corr < 0.0003) (Table 3).

**TABLE 3:** Specific (CT)*n* genotypes associated with VP resistance.

	CTSS			CTS02			CTL02		
F2	SS-S	SS-D	Fisher’s exact test	SL-S	SL-D	Fisher’s exact test	LL-S	LL-D	Fisher’s exact test
	2	78	3.40 × 10^−6^	16	64	1	30	50	3.28 × 10^−6^
	CTSS02			CTS03			CTL03		
F3	SS-S	SS-D	Fisher’s exact test	SL-S	SL-D	Fisher’s exact test	LL-S	LL-D	Fisher’s exact test
	12	68	1.80 × 10^−4^	24	56	0.88	38	42	1.10 × 10^−4^

S, surviving group; D, Dead group. Bonferroni corrections: *p corr < 0.0167 (0.05/3), **p corr < 0.0033 (0.01/3), ***p corr < 0.0003 (0.001/3).

We compared the bi-allelic genotype distribution between the survive and dead groups and shrimp with the LL genotype in the 5′-UTR of the *LvIRF* gene were resistant to VP, while those with SS were susceptible to VP. The above results suggested that shrimp with a long (CT)*n* repeat in the 5′-UTR of *LvIRF* were more resistant to VP than those with shorter (CT)*n* repeats.

### 3.5 Short CT microsatellite genotypes inhibited the growth traits of shrimps

**FIGURE 5:**
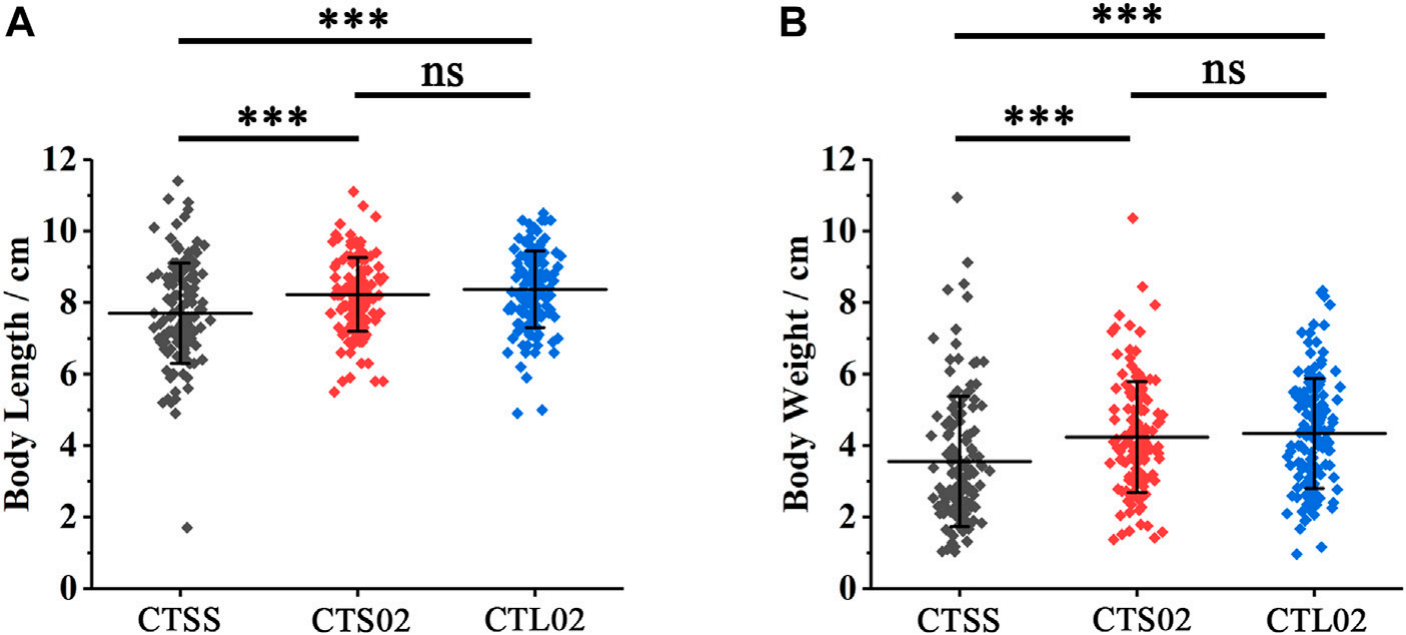
Differences in growth traits between shrimp populations in F2. **(A)** Analysis of body length differences among the three populations in F2. **(B)** Analysis of body weight differences among the three populations in F2. ***p < 0.001, ns, not significant.

On comparing body length and body weight among the three populations of F2, we found that the growth rate of the CTSS population was considerably lower than those of the CTS02 and CTL02 populations (Figure 5). In the CTSS population, 135 shrimps had an average body length of 7.70 cm and an average weight of 3.55 g. In the CTS02 and CTL02 populations, 136 and 142 shrimps were measured and counted, with an average body length of 8.22 cm and 8.37 cm and an average body weight of 4.24 g and 4.34 g, respectively. T-text analysis results show that the growth rate of the CTSS population was considerably lower than that of the CTS02 and CTL02 populations, but the growth rates of the CTS02 and CTL02 populations were not significantly different (Figure 5). The above results suggest that the shrimp with the homozygous LvIRF-SSR SS genotype may inhibit shrimp growth.

## 4 Discussion

Our study examined the role of an SSR with a (CT)*n* motif in the 5′-UTR, its repeat length variation and the association of its genetic variations with virus and bacteria resistance. Several studies have suggested that SSR repeats exist in abundance in eukaryotic genes; untranslated coding regions (UTRs) have more SSRs than coding regions and exhibit a strong bias towards di- and tri-nucleotides repeats (Li et al., 2002; Fujimori et al., 2003; Zhao et al., 2014). The SSRs are present in the UTRs, indicating that they are likely involved in regulating target gene transcription (Simpson and Ayyar, 2008; Colak et al., 2014). Previous research has shown that the length of the SSR in *LvIRF* can influence the expressional level, and polymorphisms of LvIRF-SSR are associated with WSSV resistance traits in shrimp (Yin et al., 2019). In this study, our goal was to investigate the application of LvIRF-SSR in improving the antiviral traits in shrimp. Genotyping selects individuals for cross-matching, compared the differences in resistance to virus of different populations and found that shrimp with smaller numbers of (CT)*n* repeats exhibited enhanced tolerance to virus infection.

In aquatic breeding, molecular markers have been used to associate with and improve biological traits. Applications of marker-assisted selection for disease resistance included the breeding of a Japanese flounder (*Paralichthys olivaceus*) strain with resistance to the viral disease lymphocystis (Fuji et al., 2006; Fuji et al., 2007), and based on molecular markers affecting resistance to bacterial cold-water disease in rainbow trout (*Oncorhynchus mykiss*) (Liu et al., 2018). Another well-known example is the major molecular markers affecting resistance to the infectious pancreatic necrosis (IPN) virus in Atlantic salmon by QTL; marker-assisted selection significantly prevented IPN outbreaks (Houston et al., 2008; Moen et al., 2009). In addition, several molecular markers associated with resistance to pancreas disease (Gonen et al., 2015) and cardiomyopathy syndrome have been used in salmon breeding (Boison et al., 2019; Hillestad and Moghadam, 2019). In this study, we used the LvIRF-SSR as a molecular marker to establish shrimp breeding programmes with diverse genotypes and tolerances to viral infection. The population of shrimps containing short CT genotypes had a stronger antiviral immune response, as manifested by the observation of increased *LvIRF* gene expression and prolonged survival time after WSSV and DIV1 infection. In addition, the resistance to virus could be stably passed on to offspring by marker-assisted selection. Thus, LvIRF-SSR could be a candidate marker for shrimp breeding for virus resistance.

Furthermore, we noted that the CTSS population is resistant to viruses while susceptible to bacteria (VP). Previous studies have shown that activating the antibacterial NF-κB pathway in *L. vannamei* benefits the transcription and replication of WSSV genes (Qiu et al., 2017; Li et al., 2019a). Host transcription factors—Dorsal, Relish, c-Jun and c-Fos—have all been shown to regulate a bulk of viral genes, such as wsv069, wsv079 and wsv249 (Huang et al., 2010; Li et al., 2015b; Li et al., 2016). Meanwhile, the antiviral STING-IRF-Vago-JAK/STAT pathway can negatively regulate the activity of the NF-κB pathway (Li et al., 2019b). The transcriptional activity of microRNA-1 (miR-1) molecule from *L. vannamei* could be regulated by the JAK-STAT pathway, with miR-1 serving as the mediator, and STAT could exert a negative regulatory effect on the Dorsal pathway (Zuo et al., 2020). In other words, resistance to virus and bacteria in *L. vannamei* may have antagonistic effects; shrimps with strong resistance to virus are susceptible to bacteria, while those with strong resistance to bacteria are susceptible to virus. In our study, we investigated the bi-allelic genotype distribution and phenotypic variations in two generations of offspring. Through associated analysis and artificial challenge, we found that shrimps with the LL genotype in LvIRF-SSR were resistant to VP and susceptible to WSSV, while those with the SS genotype in LvIRF-SSR were resistant to WSSV and susceptible to VP. The LvIRF-SSR marker in selective breeding can be used to improve antiviral or antibacterial traits.

SSRs are co-dominant Mendelian markers, and they can inherit in accordance with Mendelian inheritance (Powell et al., 1996). In sugarcane (Lu et al., 2015), *Pseudotsuga menziesii* (Slavov et al., 2004) and peanut (Zhou et al., 2016), the segregation ratios of the SSR markers were consistent with the Mendelian inheritance law for single loci. However, our investigation discovered that the LvIRF-SSR of *L. vannamei* did not segregate in a Mendelian ratio of 1:2:1, but rather as a phenomena of homozygous deletion in offspring. We speculated that homozygous deletion might relate to bacterial susceptibility and slow growth of the genotype SS in shrimps. The CTSS population was susceptible to bacteria and grew more slowly compared to the CTS and CTL populations, indicating that shrimps with the SS allele are more likely to be eliminated in natural environments. This also explains the inability to segregate the SS allele of shrimp in the natural population when breeding the F1 generation.

In summary, we identified that the LvIRF-SSR marker could be used as a molecular marker in shrimp breeding. By cross-matching, enriching the dominant allele in offspring can improve the antiviral or antibacterial traits. Our results provide some insights into how this SSR could be used as a molecular marker in shrimp breeding.

## Data Availability

The original contributions presented in the study are included in the article/Supplementary material, further inquiries can be directed to the corresponding authors.
